# School nurses’ perceived capability, opportunity, and motivation to provide health promotion: A convergent mixed-methods study

**DOI:** 10.1016/j.ijnsa.2025.100396

**Published:** 2025-08-05

**Authors:** Malin Jakobsson, Marianna Moberg

**Affiliations:** a Department of Nursing, School of Health and Welfare, Jönköping University, Jönköping, Sweden; b CHILD-research group, Jönköping University, Jönköping, Sweden; c Department of Neurobiology, Care Science and Society, Karolinska Institutet, Stockholm, Sweden

**Keywords:** Nurse, Health promotion, Interprofessional teams, COM-B, Capability, Opportunity, Motivation, Mixed-methods

## Abstract

**Background:**

Schools are described as important arenas for health promotion interventions. Despite the critical role of school nurses in health promotion, limited research specifically examines school nurses’ perceptions of providing health promotion.

**Aim:**

We aimed to describe both ratings and descriptions of school nurses' capability, opportunity, and motivation to provide health promotion beyond the individual health dialogues in Swedish schools.

**Method:**

This convergent mixed-methods study used national cross-sectional data collected in May 2023 from a web-based survey and qualitative narrative data from an open-ended question in the same survey. In total, 596 school nurses in Sweden answered the web survey, and 354 described their experiences in the open-ended question. Data were analysed and interpreted through three commonly used theoretical domains in behaviour change research: capability, opportunity, and motivation.

**Results:**

School nurses’ perceived motivation and capability to provide health promotion were generally rated as high, whilst the perceived opportunities to provide health promotion was rated as lower. Individual factors and the local work environment seemed to influence school nurses' perceived capability, opportunity, and motivation to provide health promotion. In a positive work environment, school nurses were confident in colleagues’ expectations and how to assess health promotion needs. In addition, there was a joint agenda, structure, and interprofessional collaboration in such a school, where health promotion felt important, enjoyable, and rewarding. In a negative work environment, school nurses were not expected to provide health promotion, and their time was prioritised to include other tasks primarily. Moreover, there was a lack of perceived adequate knowledge, skills, and tools to provide health promotion.

**Conclusion:**

A positive work environment for school nurses, including a joint health promotion agenda, interprofessional collaboration, and clear expectations and structure, seem to be essential for providing health promotion activities to school-aged children.

**What is already known**

• Schools and school health services are expected to provide health promotion.

• School nurses feel overwhelmed with health promotion needs.

Alt-text: Unlabelled box

**What this paper adds**

• The work environment in schools was perceived as both a barrier and a facilitator for health promotion activities.

• School nurses requested a joint health promotion agenda and evidence-based activities.

• Interprofessional collaborations and clear expectations were important for a positive work environment.

Alt-text: Unlabelled box

## 1 Introduction

Health promotion is defined by the World Health Organization (Nutbeam, 1998) as *“…the process of enabling people to increase control over, and to improve their health*” (p. 351). Schools are described as important arenas for health promotion interventions (World Health Organization, 2021), and school nurses play a key role in promoting health and well-being among school-aged children (UNESCO, 2024). In Sweden, all children enrolled in primary, secondary, and upper-secondary schools are entitled to have continuous access to school health services, including medical, psychological, social, and pedagogical expertise, to ensure all school children reach curricular goals (Swedish Code of Statutes, 2011). In addition, all school children have a legislated right to regular health visits to a school nurse throughout the school years (Swedish Code of Statutes, 2011). Such individual health visits should include health education, prevention, and early intervention through, for example, monitoring of growth and development, immunisations, visual and hearing screenings, and individual health dialogues about healthy habits and social well-being (Swedish National Board of Health and Welfare [Swe: Socialstyrelsen] and Swedish National Agency for Education [Swe: Skolverket], 2016). Beyond the individual health visits, school health professionals are also expected to work strategically with health promotion on group and school levels such as: health education, social climate, physical environment, and student involvement (Swedish National Board of Health and Welfare [Swe: Socialstyrelsen] and Swedish National Agency for Education [Swe: Skolverket], 2016).

In contrast to other primary health contexts, the work environment of school nurses is a unique health care setting. The school nurse provides health care in an educational context and is often the only professional in this role, where the interprofessional environment typically consists mainly of pedagogical staff, such as teachers, special needs educators, and principals (Reuterswärd and Hylander, 2017). Recently, researchers have shown that school nurses seem motivated to provide health promotion. Nonetheless, the interprofessional work environment in schools can lead to school nurses experiencing challenges in providing health promotion because of discrepancies between the perceived health and educational needs of students (Flodin et al., 2024). Health promotion at the group and school level is a relatively new task beyond the health promotion school nurses do via individual health dialogues and is sparsely researched.

### 1.1 Theoretical framework

Effective interventions, such as providing health promotion activities, rely on successful implementation (Brownson et al., 2023). The complexity of human behaviours adds another layer of challenge to the implementation process. To address these challenges, the theoretical framework *behaviour change wheel* – also known as the *capability, opportunity, motivation,* and *behaviour* model – was developed by Michie and colleagues as a framework for theorising drivers of behaviour (Michie, van Stralen and West, 2011). The cornerstones of the behaviour change wheel are that a person’s perceived *capability, opportunity*, and *motivation* predict a persons’ *behaviour.* Also, the predictive *capability, opportunity*, and *motivation* domains are further divided into six sub-domains linked to the main structures of the model. *Capability* refers to the individual’s physical and cognitive ability to perform a behaviour. *Opportunity* refers to how the social and physical environment impacts behaviour. *Motivation* refers to the willingness, intentions, beliefs, and professional stance to engage in a behaviour (Michie, van Stralen and West, 2011).

For example, when implementing new protocols in diverse complex organisations, such as schools and hospitals, individuals’ perceived capability, opportunity, and motivation may be of importance. This has been shown in previous research guided by the behaviour change wheel with school nurses providing immunisation (Flood et al., 2023), school teachers providing behaviour support (Fohlin et al., 2023), and emergency nurses implementing Safeward Protocols (Yap et al., 2024).

In this paper, we have applied and referred to the applicable domains of the behaviour change wheel, *capability, opportunity*, and *motivation,* with the notion that these traits all affect school nurses' behaviour; i.e., to provide health promotion activities as expected in national statutes (Swedish Code of Statutes, 2011; Swedish National Board of Health and Welfare [Swe: Socialstyrelsen] and Swedish National Agency for Education [Swe: Skolverket], 2016). Understanding school nurses’ perceived capabilities, opportunities, and motivation to provide health promotion should be crucial for future design and successful implementation of school-based interventions. Researchers who specifically examine experienced school nurses’ perceptions of providing health promotion are few. Therefore, in this mixed-methods study, we aimed to describe both ratings and descriptions of school nurses' capability, opportunity, and motivation to provide health promotion beyond the individual health dialogues in Swedish schools.

## 2 Methods

### 2.1 Design

To address the aim of the study, a descriptive convergent mixed-methods study design (Creswell and Creswell, 2023) was used. National cross-sectional data from a web-based survey and qualitative narrative data from an open-ended question in the same survey constituted the analysed data.

### 2.2 Data collection

In May 2023, the Swedish National Association of School Nurses organized an annual conference, which that year attracted 1500 attendees, about half of the most recent (2022) estimate of the number of school nurses working in Sweden (Swedish Government Official Reports (SOU 2021:11), 2021). In connection with the annual conference, the conference visitors were invited to respond to an anonymized electronic survey. One day before the conference, written information about the study purpose and contact information, together with a link to the survey, were e-mailed to all members of the Swedish National Association of School Nurses (*n =* 2400), and the link was also published on two of the association's public social media channels (Facebook and Instagram). Conference visitors (*n =* 1500) were encouraged to respond through printed posters with QR-coded links and reminded occasionally from the stage to participate in the survey. Answers only from those school nurses who had been active during the previous school year were used in analyses.

### 2.3 Participants

In total, 596 school nurses answered the web survey, and 354 described their experiences in the open-ended question (Table 1). School nurses representing all of Sweden’s 21 regions participated in the survey. The average age of the respondents was 50 years old, and almost all identified as women. When converting reported hours of employment to a monthly equivalent, school nurses working full-time (40 h per week) were responsible for an average of 491 children each.

**Table 1:** Characteristics of participating school nurses (*N* = 596).

	%
**Background**	
Sex: Woman	98.8
Age	49.6 A
Students^1^	491 A
Employment scope^2^	90
Schools	2.6 A
**Years as a school nurse**	
2 years or less	18.6
3 to 5 years	24.7
6 to 10 years	27.3
11 to 20 years	21.2
21 years or more	8.2
**Child age (grades)**	
6–13 years (0–6)	34.4
6–16 years (0–9)	22.3
10–16 years (4–9)	5.7
14–16 years (7–9)	10.9
17–19 years (10–12)	18.8
Other combinations	7.9
**School organisation**	
Public	82.4
Private	14.6
Both private and public	3.0
**Employer**	
School principal or equivalent	55.4
Coordinator of centralized school health service at the municipality	40.6
Business corporate or self-employed	4.0

A = average.

1 . Number of students per full-time (40 h per week).

2 . Employment scope expressed as percent per full-time (40 h per week).

School nurses working with children aged 6–19; i.e., in primary, secondary, and upper secondary schools, participated in the survey. Most respondents worked at a public school with students younger than 16. A majority of school nurses declared that they were employed by the school principal, while some were centrally employed; e.g., by a municipal school health coordinator.

### 2.4 Data

#### 2.4.1 Quantitative data

Survey items were developed and adapted by the research team to reflect school nurses' perceived capability, opportunity, and motivation to provide health promotion activities in their everyday work environment. Responses were on a 5-point Likert scale. The survey was initially pilot tested with board members (*n* = 10) of the Swedish National Association of School Nurses. The board members were asked to focus on comprehensibility of the item formulations and on the survey content; i.e., if the questions were clear and easy to understand and if any crucial aspects of the theoretical domains were missing in relation to school nurse practice. Written feedback was gathered, and the board members approved revisions to the items.

#### 2.4.2 Qualitative data

The web survey posed an open question to the respondents. The question was phrased in Swedish and translated in English as: *Please describe your health promotion work as a school nurse, beyond the individual health dialogues, in your own words*. The answers ranged from 2–576 words, most of which were between 100–200 words.

### 2.5 Convergent mixed-methods analysis

Convergent mixed-methods analyses were performed in three analytical phases. First, quantitative and qualitative data were analysed separately with appropriate methods. Second, the two databases were merged in an integration analysis, and third, meta-inferences were abstracted and displayed in a joint display of findings (Creswell and Creswell, 2023).

The reporting checklists Strengthening the Reporting of Observational Studies in Epidemiology (STROBE) (Von Elm et al., 2007) and the Consolidated framework for Reporting Qualitative research (COREQ) (Tong, Sainsbury and Craig, 2007) were followed.

#### 2.5.1 Quantitative analysis

The initial phase of the integrative data analysis was to analyse statistically school nurses’ ratings of perceived capability, opportunities, and motivation to provide health promotion (Table 2). First, the construct validity of the final items (*n =* 9) was assessed with confirmatory factor analysis. Internal consistency was based on the mean values of the included items (Creswell and Creswell, 2023). A three-factor solution showed good item loadings (>0.7), with acceptable internal consistency (α = 0.71 to 0.82) and in agreement with the theoretical framework (Michie, van Stralen and West, 2011). To enable descriptive analyses, the mean scores of each factor (Capability, Opportunity, Motivation) were categorized into three levels: high (mean ≥3.6), neutral (mean *=* 2.6 to 3.5), and low (mean ≤ 2.5).

**Table 2:** Description of factors, items, factor loadings, and internal consistency (*n* = 596).

	**Factors and items**	**Mean (SD)**	**Factor loading**
	**Capability (α = 0.78)**	4.1 (0.7)	
Q1	I feel confident providing health promotion in larger groups of students	4.2 (1.0)	.71
Q2	I have updated topic knowledge to provide health promotion	4.1 (0.9)	.88
Q3	I have adequate topic knowledge to provide health promotion	4.2 (0.8)	.86
	**Opportunity (α = 0.82)**	3.8 (0.8)	
Q4	My school has a well-functioning student health organization that collaborates regarding in providing health promotion	3.8 (1.1)	.83
Q5	My employer encourages me to provide health promotion	4.1 (1.1)	.83
Q6	My employer has a clear strategy for providing health promotion	3.4 (1.1)	.86
	**Motivation (α = 0.71)**	4.6 (0.5)	
Q7	Health promotion contributes to health equity among the students	4.5 (0.6)	.81
Q8	There are clear benefits for the students if I provide health promotion	4.6 (0.7)	.80
Q9	Providing health promotion encourage my professional creativity	4.6 (0.7)	.73

SD = Standard Deviation*; α* = Cronbach's alpha, internal consistency of construct

Note: Lickert scale: 1 (strongly disagree) to 5 (strongly agree).

#### 2.5.2 Qualitative analysis

The narrative data were analysed using qualitative content analysis with a deductive manifest approach (Graneheim and Lundman, 2004; Lindgren, Lundman and Graneheim, 2020). The three domains, capability, opportunity, and motivation, deriving from the behaviour change wheel (Michie, van Stralen and West, 2011), were used as an analytic framework in accordance with the study aim. In the first step of the analysis process, the data in the form of the original transcript were read repeatedly in their entirety. In the second step, the data were de-contextualized into meaning units: words and sentences pertaining to the domains were marked and lifted out of the text into a new document with a table where all meaning units were collected. In the third step, the meaning units were condensed and coded. Coding means labelling the condensed meaning unit with a descriptive code close to the original text and on a low level of abstraction and interpretation (Graneheim and Lundman, 2004; Lindgren, Lundman and Graneheim, 2020). To decrease the risk of missing essential content, the original transcript was read in parallel to ensure its content was preserved, which is an aspect of credibility. After that, the codes were carefully sorted into groups based on similarities and differences during re-contextualization. Finally, after an abstraction and interpretation of the text, 10 subcategories and three main categories emerged while maintaining the manifest content. The analysis process moved back and forth between the original text and its parts to ensure trustworthiness. The authors have worked side by side and recurrently processed and discussed the analysis, subcategories, and main categories. Quotes are used in the result to ensure credibility (Lindgren, Lundman and Graneheim, 2020).

#### 2.5.3 Integration analysis and meta-inferences

The second phase of the integrative data analysis was to compare the two databases in an integrated analysis (Creswell and Creswell, 2023). Here, the statistical and qualitative findings were brought together for further integrated analysis and comparison in a joint display of findings (Fetters et al., 2013). During the third phase of analysis, which included creating the joint display and new inferences, the researchers engaged in a creative approach with the intention of further understanding the datasets and illustrating meta-inferences (Guetterman, Fetters and Creswell, 2015; Creswell and Creswell, 2023).

### 2.6 Ethical considerations

We followed the ethical regulations and guidelines outlined in Swedish law, where no ethical approval from the national ethical review authority was needed when no sensitive personal data, such as birth date, religion, race, or health status, is requested, and when the study does not involve an intervention that aims to affect the participant physically or psychologically (SFS, 2003:460). The authors have sought ethical approval from the national ethics review authority regarding a similar project, where data collection took place in the same way via a questionnaire during a school nursing conference. Then, the statement from the ethical review authority concluded that no official ethical approval is needed for such research, according to the Swedish law (SFS, 2003:460). An ethical self-review within the academy and the research team was conducted, and the code of ethics was carefully followed by fulfilling the requirements of respect for autonomy, nonmaleficence, beneficence, and justice (Swedish Research Council [Swe: Vetenskapsrådet], 2024).

Considerations were applied to ensure participant autonomy and safety; e.g., written information stating what participation in the study means and allowing the individual to decide for themselves whether they want to participate. In the study, all data was anonymized; i.e., no personal information, such as name, municipality, school, e-mail, or IP address was collected, and responses were securely treated and stored. At the beginning of the survey, the school nurses made an active agreement to participate (yes or no). The school nurses were also informed about how the results of the study would be published and that it would be presented to them as a profession for their benefit.

## 3 Results

We have described, in a mixed-methods approach, both ratings and descriptions of school nurses' capability, opportunity, and motivation to provide health promotion in primary, secondary, and upper secondary schools in Sweden, beyond the statutory individual health dialogues.

### 3.1 Ratings of providing health promotion

The participants generally rated their motivation to provide health promotion as high, while their perceived capability was rated slightly lower, and opportunities the lowest (Table 3). Respondents reported the greatest confidence in their motivation to provide health promotion, as almost all participants rated their motivation as high. In contrast, perceived opportunities to provide health promotion showed greater ambiguity, with more neutral and low ratings reported.

**Table 3:** Ratings of perceived capability, opportunity, and motivation to provide health promotion (*n* = 596).

	**Capability**	**Opportunity**	**Motivation**
Mean	**4.1**	**3.8**	**4.6**
High ≥ 3.6 ( %)	83.6	65.1	97.1
Neutral 2.6–3.5 ( %)	13.3	25.2	2.0
Low ≤ 2.5 ( %)	3.2	9.7	0.8

*Note*: Lickert scale: 1 (strongly disagree) to 5 (strongly agree).

### 3.2 Descriptions of providing health promotion

The qualitative analysis yielded 10 factors (i.e., sub-categories) influencing the participants' health promotion work (Table 4). The factors were divided into three overarching categories based on the behaviour change wheel domains (capability, opportunity, and motivation).

**Table 4:** Overview of domains, main categories, and subcategories representing school nurses' capability, opportunity, and motivation in implementing health promotion work at the group and school levels.

**Domains**	**Main categories**	**Subcategories**
**Capability**	The health promotion activities depend on the individual's capabilities	Having work experience bring benefits
		Lacking skills or self-efficacy create barriers

**Opportunity**	The opportunities for health promotion are enormous but not prioritized	Knowing all the students in the school is a unique advantage
		Having well-functioning cooperations, internally and externally, increase the chances of health promotion.
		Lacking time brings unrealistic conditions for providing health promotion
		Unsupportive leadership and lack of structure makes it complicated

**Motivation**	The motivation to provide health promotion is stimulated and restrained by internal and external factors	Being a significant adult and creating trusting relationships creates engagement
		Guiding students to healthy choices and self-care generates gratitude and job satisfaction
		Feeling resigned when colleagues do not see the benefits of health promotion
		Having an opinion that school nurses should not engage in health promotion at the group and school levels reduces engagement

#### 3.2.1 The health promotion activities depend on the individual's capabilities

The participants experienced that developing health promotion activities depended on the capability of the individual participant. Therefore, their work experience was beneficial. At the same time, the work was more challenging if the participants lacked skills or self-efficacy.

**Having work experience bring benefits**

Participants felt that work experience gave them confidence, security, and competence, which were invaluable when developing health promotion work. Their expertise helped them not only to prioritize acute issues but also to plan for the long term and take a helicopter view. The sense of security it provided also led to a permissive approach toward themselves; if a planned health promotion session did not work as intended the first time, they felt free to be creative and try a different variant next time. The participants also described how their experience gave them confidence in listening to what this individual or class needed in order to promote health.

“…based on my work experience and professional confidence (as a school nurse), I am able to develop my role towards providing more health promotion and not only end up in emergency measures…” (59)

**Lacking skills or self-efficacy create barriers**

One experience that participants shared was that health promotion depended on their skills, willingness, and interest. As new school nurses, they might not have known how they could or should work to promote health. It was also explained that school nurses did not possess the pedagogical skills teachers had for presenting in classes, and this could make it challenging to carry out health promotion at the group and school levels. To strengthen their ability to do so, they expressed a desire for clearer guidelines, instructional materials, and recurring training.

“…I try to do my best regarding students' needs for support in different health issues, but I want to deepen my knowledge and provide real help. It takes time to learn, but I look forward to feeling confident in the health promotion work…” (164)

#### 3.2.2 The opportunities for health promotion are enormous but not prioritised

Participants described that opportunities to promote health in schools as enormous. Through individual health dialogues with all students, they had access to an invaluable source of information that could guide health promotion efforts toward the most pressing issues within a group or the school as a whole. With well-functioning collaboration among colleagues in school health services, teachers, and external partners, the potential for impactful health promotion was seen as limitless. However, the opportunities did not align with the conditions provided. A lack of time, unsupportive leadership, and inadequate structures made the work feel unrealistic.

**Knowing all the students in the school is a unique advantage**

Participants said they were the only adults in the school setting who regularly met with all students through individual health dialogues. The insight they gained into students' health status and needs was invaluable. When the school nurse summarized the outcomes of the health dialogues, the needs of the group, or school could be clarified. Furthermore, they experienced that the opportunity to promote students' health increased when efforts were made at the individual, group and school levels. The school nurse could deepen reflections with all students in the class, and the individual and group conversations enriched one another.

“…The health dialogues with the health surveys provide a good picture of the health situation at the group level…and the possibility of targeted interventions in classes…” (349)

**Having well-functioning cooperations, internally and externally, increase the chances of health promotion**

Working on health promotion at the group and school level was described as a team effort, in which school health professionals collaborated with each other and with teachers. These collaborations — with counsellors, special educators, psychologists, and teachers — included health-related lessons and group work. Topics could include values, puberty, sexual health, school absenteeism, diet and exercise, sleep, alcohol and drugs, safety, bullying, and other relevant health-related issues that needed to be addressed in class. Internal collaboration was seen as crucial to the success of health promotion in schools. In addition, participants found external collaboration with youth clinics, social services, leisure services, and other organisations valuable. Sometimes, external resources were needed to fully address the student´s overall situation.

“…The counsellor and I, but also the rest of the school health services, pick up on needs and try to create health promotion. It is a team effort…” (72)

**Lacking time brings unrealistic conditions for providing health promotion**

A recurring experience among participants was that time for conducting health promotion work at the group and school levels was scarce or non-existent. Most participants described that the statutory task of conducting individual health dialogues with students consumed all their available time. Furthermore, it was explained that the extent of health promotion work at the group and school levels varied across the country; some participants had the time, while others did not. This, in turn, led to unequal care for children and adolescents nationwide – something school nurses found deeply frustrating. The lack of time, combined with both their own and others' expectations to fulfil this task, created unrealistic work conditions.

“…If I am to work more at the group and school level, I need to have far fewer students. I do not have time as the situation is today…” (173)

**Unsupportive leadership and lack of structure makes it complicated**

Participants called for clear leadership to enable well-functioning and systematic health promotion work. They emphasized the importance of principals, who were responsible for both students' education and health, having the necessary knowledge, prioritizing health promotion, and creating the conditions for it to succeed. Furthermore, participants felt that a structured and systematic approach to health promotion would benefit both themselves as providers and the students as recipients. There was also a strong demand for national guidelines to support health promotion efforts, including individual health dialogues, as well as work at the group and school levels.

“…Unfortunately, there is no clear structure from management and supervisors to carry out the health promotion work as I would have wished…” (539)

#### 3.2.3 The motivation to provide health promotion is stimulated and restrained by internal and external factors

The participants described that internal and external factors influenced their motivation to engage in health promotion work at the group and school levels, beyond the statutory individual health dialogues. Their motivation increased when they recognized their role as significant adults who built trusting relationships with students and when they saw that their guidance led to healthy choices and improved self-care. Motivation was lower among participants who believed that health promotion work at the group and school levels was not part of their role or when colleagues did not see the value of such work.

**Being a significant adult and creating trusting relationships creates engagement**

Participants experienced that providing health promotion at the group and school levels meant meeting students in contexts beyond the statutory individual health dialogues held in the school nurse's office. In these settings, they could see students from a different perspective and observe their social interactions. It was also described as enjoyable to meet curious students in group settings. In the classroom, school nurses and students got to know each other better, which was perceived to strengthen their relationship. As a result, students were more likely to speak openly and show vulnerability during individual health dialogue. The participants' sense of being important adults with meaningful relationships with students increased their motivation and engagement in health promotion at the group and school levels.

“… It is important for me to be a safe adult at school and build relationships with students. It makes it easier for me to raise sensitive and difficult issues when I have a connection with the students, and they see me as someone familiar rather than a stranger…” (470)

**Guiding students to healthy choices and self-care generates gratitude and job satisfaction**

Participants described a profound sense of gratitude, honour, and job satisfaction when they were involved in guiding children and adolescents toward better health. They expressed joy in supporting students to make healthy choices that could benefit them now and in adulthood. Topics raised during the individual health dialogues could also be discussed more generally in class or in small groups, allowing health-related issues to be addressed in different ways. For example, if many students in a class experienced sleep problems, increased sedentary behaviour, or skipped lunch, these issues could be explored collectively in group settings. The knowledge that their efforts contributed to healthier choices and improved self-care gave school nurses a strong sense of fulfilment, which in turn increased their motivation to engage in health promotion at the group and school levels.

”… It feels good to give students knowledge and insight that they themselves can largely influence their well-being through a good lifestyle…” (42)

**Feeling resigned when colleagues do not see the benefits of health promotion**

Participants described a lack of motivation when colleagues did not recognize the value of health promotion work at the group and school levels. The knowledge they had gathered about the health situation in classes, through the individual health dialogues, was often not acknowledged or given space for joint reflection within the school health services team. This led to feelings of being minimized as professionals. In addition, school leadership and teachers sometimes failed to see how prioritizing certain health-related issues in the classroom could benefit students' learning.

“…The hardest part is getting other school staff to understand the importance and magnitude of our health promotion work…” (290)

**Having an opinion that school nurses should not engage in health promotion at the group and school levels reduces engagement**

Participants felt that health promotion was most effectively carried out through individual health dialogues. They expressed that the available time should be devoted entirely to these dialogues and to systematic follow-ups. The perception was that school nurses were trained and intended for personal interactions with students and that they had possessed specific skills in motivational interviewing. Teaching general health in classroom was seen as the responsibility of teachers. Beliefs that health promotion at the group or school levels was not beneficial contributed to reduced motivation to engage in such work.

“…I am convinced that individual health dialogue is what we should focus on, not groups…” (574)

### 3.3 Joint display of ratings and descriptions

An integrative analysis of ratings and narrative descriptions resulted in a joint display of findings (Table 5). School nurses generally rated their motivation and capability to provide health promotion as high, while their perceived opportunities to do so were rated lower. The narrative described motivation for health promotion as foundational, an important part of their professional identities. Being a significant adult and playing a key role in the health and development of school-aged children brought feelings of joy and satisfaction. Capability was described in terms of feeling safe and secure, grounded on knowledge, skills, and work experience. However, school nurses also highlighted a lack of opportunities, often feeling misunderstood in their professional role by pedagogical colleagues and constrained by limited time to plan and implement health promotion activities beyond the regular health dialogues.

**Table 5:** Joint display of findings, with ratings and perceptions of capability, opportunity, and motivation.

**Rating**	**High**		**Neutral**		**Low**	
**Capability**	**83.6**	*“…The school nurse can use her skills and experience in every encounter with individual pupils, groups of pupils, guardians, and other school staff…”* (66)	**13.3**	*“… I'm quite new as a school nurse. I'm constantly trying new approaches and exercises to promote health; some things work, others don't, and I adjust according to what has worked well …”* (81)	**3.2**	*“…I try to do my best regarding students' needs for support in different health issues, but I want to deepen my knowledge and provide real help. It takes time to learn, but I look forward to feeling confident in the health promotion work…”* (164)
		*“…Being accessible, hopeful, and listening is my approach, I feel confident with health promotion …”* (159)		*“… It works, but I think school nurses need more tools and training to develop the health promotion work…”* (309)		*“…It is also desired that the school nurse is involved in the educational aspect where I have sufficient knowledge…”* (121)
		*“…based on my work experience and professional confidence (as a school nurse), I am able to develop my role towards providing more health promotion and not only end up in emergency measures…”* (59)		*“… We are now undergoing training to improve student health; hopefully, this will make us work more to promote health and not mostly put out fires …”* (137)		*“… Unfortunately, training, further education, and development are non-existent …”* (298)

**Opportunity**	**65.1**	*“…The health dialogues with the health surveys provide a good picture of the health situation at the group level…and the possibility of targeted interventions in classes…”* (349)	**25.2**	*“… If there were national programs to implement at group/organization level, it would simplify …”* (178)	**9.7**	*“…If I am to work more at the group and school level, I need to have far fewer students. I do not have time as the situation is today…”* (173)
		*“… Health promotional work at the group level is an important tool to reach all pupils equally, preferably in collaboration with teachers. The best is when you find ways to work interprofessional …”* (263)		*“… I consider prevention and health promotion to be very important and rewarding, but I do not have the time to do it to the extent I would like…”* (439)		*“…There is no support from the management to do it! Without support, it is not possible; it feels like the principal mostly wants you to be an emergency room at school, putting out fires instead of working preventively…”* (139)
		*“… it is perfect; we have an annual wheel with different reoccurring themes for different grades that is updated for each new school year …”* (68)		*“… it is very much based on the school nurse's interest and planning. With more clarity [in guidelines] the school nurses would become more confident in what is expected …”* (104)		*“… projects have run aground because of the management's lack of capacity to come to decisions and help to see opportunities instead of problems. I am convinced that health promotion is a matter of democracy and an important compensatory factor …”* (424)

**Motivation**	**97.1**	*“… It's one of the most fun and rewarding parts of being a school nurse. Getting more insights into the students' lives and in the classes. To influence students to make healthy choices feels incredibly important …”* (560)	**2.0**	*“… It is important, but it takes an effort to start; cooperation with other professions [in the school] is limited. We have a lack of understanding of what we can and should expect from each other…”* (435)	**0.8**	*“… [health promotion] takes time, my workload is heavy, and there are no measurable outcomes to motivate…”* (168)
		*“… It is important for me to be a safe adult at school and build relationships with students. It makes it easier for me to raise sensitive and difficult issues when I have a connection with the students, and they see me as someone familiar rather than a stranger…”* (470)		*“… I think we must remind ourselves that health promotion already occurs in the health dialogues. Surely, at the individual level [and not on group or school level] …”* (76)		*“…I am convinced that individual health dialogue is what we should focus on, not groups…”* (574)
		*“… Working on health promotion with students in different settings enriches both myself and the students …”* (337)		*“… My view of what constitutes health promotion does not always match what the principal wants me to do …”* (426)		*“…The hardest part is getting other school staff to understand the importance and magnitude of our health promotion work…”* (290)

The integrative analysis further indicated that, in a positive work environment, school nurses felt confident in their responsibilities and expectations. They were able to assess health promotion needs based on health surveys and students encounters. These environments were characterized by a joint agenda, clear structure, and strong interprofessional collaboration, making health promotion feel important, enjoyable, and rewarding. In contrast, in negative work environments, school nurses were not expected to engage in health promotion, and their time was redirected toward other tasks. These settings were marked by a lack of knowledge, skills, and tools, which further hindered their ability to carry out health promotion work.

## 4 Discussion

In this mixed-methods study, we aimed to describe both ratings and descriptions of school nurses' capability, opportunity, and motivation to provide health promotion. Data were gathered through a questionnaire that included an open-ended question. School nurse participants were asked about their capability, opportunity, and motivation to provide health promotion in schools, beyond the statutory individual health dialogues they hold with each student. We found that participants were highly motivated to provide health promotion in groups or school level. Motivation was related to internal and external factors, generating joy and satisfaction in work. Even the capability among school nurses was relatively high and often associated with the confidence that long work experience provided. In contrast, participants perceived their opportunities for health promotion to be lower. Hindrance was mainly due to a lack of time, unsupportive leadership, and inadequate structure. The three theorised drivers of human behaviours of the behaviour change wheel (Michie, van Stralen and West, 2011) – capability, opportunity, and motivation – guided the study and shed light on school nurses' perceptions and practices in health promotion. All three factors seem to have contributed to driving the school nurse's behaviour.

When it comes to psychological capability (Michie, van Stralen and West, 2011), participants strongly valued their work-experience and previously attained knowledge about children’s health. The security and calm that work experience brought were described in previous research as a key factor in school nurses' health promotional work (Jakobsson, 2024). Also, school nurses have stated that, without experience and knowledge, they experienced difficulties in initiating or feeling comfortable with health promotion work at the group or school level, particularly regarding mental illness (Flodin, Lejtzen and Gunnarsdóttir, 2024; Hoskote, Croce and Johnson, 2023), sleep (Jakobsson, 2024), honour-related violence (Harding et al., 2019), or unhealthy growth development (Skantze, Almqvist-Tangen and Karlsson, 2023). To strengthen the perceived physical capability of school nurses, such as skills in performing health promotion activities (Michie, van Stralen and West, 2011), the results point to the need for regular skills development, supervision, and a good work environment, which may contribute to school nurses remaining at their workplaces. Newly-graduated school nurses may be helped by a mentoring system. While it is partly the responsibility of the school nurse to maintain and develop relevant knowledge and skills, the lower perceived ability was an organizational issue that management must be aware of and prioritize accordingly.

Regarding physical opportunities (Michie, van Stralen and West, 2011), we have pointed out organizational conditions that hindered the opportunity to provide health promotion, such as a lack of time, structure, and management. In the present study, most participants declared responsibility for between 300–500 students, while some reported responsibility for 600–1000 students. These irregularities in workload indicated that the burden and opportunities to provide health promotion varied between participants. McKinley Yoder *et al*. (2022) showed an association between school nurses’ workload and students’ academic outcomes. This further emphasised the value of enough time for the school nurses' health promotion work. Nonetheless, there are more factors than just the number of students that affect school nurses’ workload, such as the number of school units and socio-economic areas (McKinley Yoder et al., 2022; Davis et al., 2021).

Furthermore, social opportunities should be of importance for school nurses' health promotion activities (Michie, van Stralen and West, 2011). Participants described ambiguous expectations and norms in the interprofessional team as barriers for health promotion. Example of this could be school leaders and other pedagogic colleagues not being familiar with health promotion work or not prioritizing and leading it in a favourable way. These organizational shortcomings might mean that school nurses who are motivated to provide health promotion at the group and school levels do not perceive have that opportunity. This is unfortunate, because international guidelines suggest that children and adolescents' healthy habits benefit from being established early in life and that health promotion work at the group and school levels could contribute to this (World Health Organization, 2021). Participants experienced that organizational factors hindered the opportunities to present health promotion, which is something that should be reviewed for more equal care. This means that students, regardless of which school they attend, should have access to the same level of health services. It should be kept in mind before new health promotion interventions are started.

Participants' motivation to engage in health promotion at the group and school levels was multifaceted and shaped by both automatic (internal) and reflective (external) motivational components, as conceptualized in the behaviour change wheel (Michie, van Stralen and West, 2011). Examples of internal motivators, such as emotions, perceived rewards, and personal growth, were evident in the nurses’ expressions of joy, gratitude, and professional fulfilment when building trusting relationships with students and witnessing positive behavioural changes. These intrinsic factors fostered a sense of meaning and job satisfaction, which are known drivers of sustained motivation (Deci and Ryan, 1985).

Participants' expressions of reflective motivation were linked to beliefs about role identity and their perceived potential to influence expected health outcomes. Participants who viewed themselves as important role models in the school environment described a stronger intention and commitment to engage in health promotion at the group and school levels. These participants expressed a strong belief in positive consequences of health promotion activities, leading to trusting relationships with students and at the same time guiding students toward healthier choices. These beliefs were said to reinforce a professional identity and strengthened their motivation to engage in health promotion. Conversely, participants who questioned whether health promotion at the group level belonged within their professional role or who lacked belief in its impact, expressed lower motivation—indicating how doubts about role legitimacy and goal relevance can dampen reflective motivation. Participants expressed that school nurses’ skills were best utilized in individual meetings with students, such as in health dialogues or when students sought simpler medical care. Within this group, there was no doubt that most of the school nurses' time should be allocated for health promotion in individual meetings. In line with this, previous researchers showed that students valued the individual meeting with the school nurse and appreciated the personal tips and advice they received more than those given in a classroom setting, which could be more general (Jakobsson, Josefsson and Högberg, 2022; Kostenius and Lundqvist, 2022). Still, according to Swedish regulations, health promotion in schools should be carried out on three organizational levels: individual, group, and school (Swedish Code of Statutes, 2011; Swedish National Board of Health and Welfare [Swe: Socialstyrelsen] and Swedish National Agency for Education [Swe: Skolverket], 2016). Moreover, health promotion interventions at group and individual levels seem to pollinate one another. Both students and school nurses have expressed positive reactions in previous studies about school nurses spending more time in classrooms, corridors, and canteens (Hilli and Pedersen, 2021; Gädda, Hemberg and Nyman-Kurkiala, 2023; Jakobsson, Josefsson and Högberg, 2022; Rising Holmström and Boström, 2021). Participants said that by seeing the students in the classrooms, they also gained knowledge about the student’s social interaction and classroom environment and that this is favourable knowledge to have when meeting the individual student. These insights further emphasise the need for research that supports the development of well-designed, evidence-based classroom interventions for health promotion, with effective and feasible outcomes.

If school nurses view themselves solely as providers of individual care, it is likely to reduce their motivation to initiate or maintain health promotion practices at broader levels. This illustrates how motivation not only is a function of personal emotion and satisfaction but also is tied to broader role constructions and organizational norms (Wigfield, Muenks and Eccles, 2021). Furthermore, we have demonstrated that participants perceived their motivation as lacking when colleagues did not recognise the benefits of health promotion work at the group and school levels. Also, motivation was described as being affected negatively when school nurses’ attained knowledge about health on group level was not applied or appreciated when planning for health promotion activities at the school level. When school leadership and teacher colleagues failed to recognize or support the nurse’s health promotion role, it undermined both their reflective motivation and their perceived professional legitimacy—factors that are critical for motivation and behaviour (Michie, van Stralen and West, 2011).

This ambivalence highlights a tension in the school health setting between health promotion's individual and collective dimensions. While school nurses may feel intrinsically motivated, the lack of structural support and conflicting role expectations can impede their capability to act on these motivations. As motivation is not solely an internal force but embedded in social and organizational contexts, interventions aiming to strengthen health promotion work should consider both intrapersonal drivers and systemic facilitators. Ultimately, school nurses' behaviour in health promotion is dependent on their capability, opportunity, and motivation – all of which must be supported to achieve sustainable practice.

### 4.1 Strengths and limitations

The convergent mixed-methods design employed in this study offered several strengths and limitations. By integrating quantitative and qualitative data, researchers hoped to achieve a comprehensive understanding of the research problem, capturing both numerical insights and personal experiences. This approach enhanced the validity of the findings, as the strengths of one method can compensate for the weaknesses of the other, and provides rich, contextual data that adds depth to the quantitative results. This contributes to the credibility of the study, as multiple data sources support the interpretations (Lincoln and Guba, 1985). However, the complexity of designing and implementing mixed-methods studies presents challenges and a potential limitation, requiring careful planning and coordination (Creswell and Creswell, 2023; Lincoln and Guba, 1985). Additionally, integration of the data can be challenging, necessitating expertise in both quantitative and qualitative analysis to avoid potential bias in interpretation. To strengthen confirmability, one measure to avoid bias was to develop the study in close collaboration with the board members of the Swedish National Association of School Nurses. Another measure was to use authentic quotes to illustrate qualitative interpretations. Also, both authors have extensive and prolonged knowledge of the school nursing field, since they have worked as school nurses for several years. This prolonged engagement supports both dependability and credibility, as it increases the likelihood of accurate understanding and representation of the context. The strength in that the authors are familiar with the context could also be seen as a limitation, as there is a risk of preconceptions influencing the analysis. However, this is something that the authors have been aware of and constantly reflected on during the analysis process. One additional limitation could be that the questions used for the quantitative data collection had not previously been validated. Nonetheless, confirmatory analysis indicated that the factors had an acceptable construct validity. Despite these limitations, the flexibility and holistic insights offered by a convergent mixed-methods design make the approach valuable for addressing complex research questions, such as school nurses’ perspectives and prerequisites of providing health promotion. The contextual richness and relevance of the findings also contribute to their transferability, allowing insights to potentially inform similar contexts or populations.

## 5 Conclusion

School nurses felt motivated and capable of providing health promotion when offered the opportunity. Opportunities consisted of a positive work environment, where school nurses were confident in expectations and in assessing health promotion needs. In a positive work environment, there was a joint agenda, structure, and interprofessional collaboration in the school, where health promotion felt important, fun, and rewarding. We have highlighted that school nurses perceived a lack of clear direction regarding their role in health promotion, underscoring the need for national guidelines. Additionally, workload constraints and a lack of interprofessional understanding presented significant barriers for school nurses to provide health promotion. Addressing these organizational and cultural factors could be crucial when planning for school-based health promotion. Future researcher should explore how different professional groups within the school context understand the concept of health promotion in relation to their profession and existing health promotion guidelines. Furthermore, gaining insights into how school health professionals perceive their colleagues and the dynamics within the interprofessional team is essential for the successfully providing health promotion activities for school-aged children.

## Funding

This research did not receive a specific grant from any funding agency in the public, commercial, or not-for-profit sectors.

## Data availability

The data is available from the corresponding author upon request.

## CRediT authorship contribution statement

**Malin Jakobsson:** Writing – review & editing, Writing – original draft, Visualization, Validation, Software, Resources, Project administration, Methodology, Investigation, Formal analysis, Data curation, Conceptualization. **Marianna Moberg:** Writing – review & editing, Writing – original draft, Visualization, Validation, Software, Resources, Project administration, Methodology, Investigation, Formal analysis, Data curation, Conceptualization.

## Declaration of competing interest

The authors declare that they have no known competing financial interests or personal relationships that could have appeared to influence the work reported in this paper.
